# Suppression of emission rates improves sonar performance by flying bats

**DOI:** 10.1038/srep41641

**Published:** 2017-01-31

**Authors:** Amanda M. Adams, Kaylee Davis, Michael Smotherman

**Affiliations:** 1 Department of Biology, Texas A&M University, 3258 TAMUS, College Station, TX 77843, USA

## Abstract

Echolocating bats face the challenge of actively sensing their environment through their own emissions, while also hearing calls and echoes of nearby conspecifics. How bats mitigate interference is a long-standing question that has both ecological and technological implications, as biosonar systems continue to outperform man-made sonar systems in noisy, cluttered environments. We recently showed that perched bats decreased calling rates in groups, displaying a behavioral strategy resembling the back-off algorithms used in artificial communication networks to optimize information throughput at the group level. We tested whether free-tailed bats (*Tadarida brasiliensis*) would employ such a coordinated strategy while performing challenging flight maneuvers, and report here that bats navigating obstacles lowered emission rates when hearing artificial playback of another bat’s calls. We measured the impact of acoustic interference on navigation performance and show that the calculated reductions in interference rates are sufficient to reduce interference and improve obstacle avoidance. When bats flew in pairs, each bat responded to the presence of the other as an obstacle by increasing emissions, but hearing the sonar emissions of the nearby bat partially suppressed this response. This behavior supports social cohesion by providing a key mechanism for minimizing mutual interference.

Echolocating bats perceive their world through a continuous series of self-generated echoes. This biosonar is naturally susceptible to acoustic interferences that interrupt or degrade the flow of information carried by returning echoes^1^. Solitary bats hunting prey in cluttered environments resolve ambiguities in the echo stream by quickly increasing sonar emission rates to meet situational demands^2^,^3^,^4^, but this strategy might become counterproductive in situations when ambiguities arise due to acoustic interference from nearby bats performing a similar task. If all bats followed a similar strategy, increasing emission rates to compensate for information loss due to conspecific interference would only compound the problem faced by bats echolocating in groups. Bats may avoid the situation altogether by changing trajectories, exiting the group, and hunting alone, but bats are social and known to hunt together^5^,^6^. Bats spend significant amounts of time flying in the company of other bats while commuting through forests, foraging or drinking in swarms^7^, or when entering or exiting noisy day roosts^1^,^8^,^9^. Currently there are no satisfactory, comprehensive explanations for whether or how groups of bats mitigate acoustic interference arising from the sonar pulses of other bats.

Previous research has identified a suite of vocal behaviors that can, at least, partially mitigate some forms of acoustic interference; several species display modest changes in pulse acoustics to enhance the distinction between theirs and conspecifics’ echoes or other background noises^9^,^10^,^11^,^12^,^13^,^14^,^15^,^16^,^17^. Free-tailed bats (*Mollosidae)* partially compensate for the confounding effects of acoustic interference by either calling louder or by changing the duration or frequency parameters of their echolocation pulses^11^,^12^,^18^,^19^. Shifts in spectral characteristics supporting the use of ‘jamming avoidance response’ have been demonstrated in field^11^,^19^ and lab^12^ settings. Like many bats, however, free-tailed bats emit short broadband multi-harmonic sonar pulses, and the relatively minor acoustic changes, so far documented, don’t provide a true escape from signal overlap (e.g.^16^), although they may facilitate cognitive mechanisms for distinguishing theirs from another bat’s echoes. Under any circumstances, the efficacy of changing pulse acoustics to escape interference from other bats is constrained by the fact that these mechanisms provide poor solutions for any except pairs or small groups of bats and are therefore likely to comprise just one part of a larger cohesive strategy for improving sonar performance when flying in groups. There is evidence that some bats may cease calling and eavesdrop to exploit signals of their conspecifics^20^, and some species may show no compensatory behaviors at all beyond responding to nearby conspecifics as they would any other source of clutter or object in their field of view, by decreasing inter-pulse intervals while shortening pulse durations and increasing bandwidth^16^,^21^,^22^. Clearly, other more comprehensive answers are needed to explain how bats mitigate interference with echolocation in large groups. Identifying how bats achieve this is important not only because of the ecological implications, but also because bat biosonar appears to perform far more robustly in noisy cluttered environments than any current man-made sonar or radar systems.

An alternative to modulating pulse acoustics is modulating the timing of their call emissions to minimize temporal overlap with another bat’s echolocation pulses^23^,^24^. Temporal strategies have been described in other animal communication systems where animals compete to transmit signals, including insects, fish, frogs, birds and mammals^25^,^26^,^27^,^28^,^29^,^30^,^31^,^32^,^33^,^34^, several of which offer clues to how bats might coordinate their pulses in time. Gymnotiform pulse-type electric fishes use a steady stream of electric discharges to electrolocate. When these fish encounter the signals of another fish, they transiently modulate their emission rates to minimize temporal overlap with the neighboring fish’s signals^32^,^35^. These brief accelerations/decelerations generate phase-differences between the neighboring fish’s signals, thereby avoiding a continuous series of overlapping emissions that would otherwise seriously degrade electrosensing for both.

Chorusing frogs attract mates by calling repeatedly at regular intervals for long periods of time. Like electric fish, frogs that detect an overlap between theirs and a neighbor’s call will briefly postpone their next call to induce a phase lag between theirs and their neighbor’s subsequent calls^26^,^30^,^34^. Our previous work with perched Brazilian free-tailed bats (*Tadarida brasiliensis)* revealed that they behave similarly to electric fish and chorusing frogs in that they often postponed call emissions after hearing the pulse of a nearby conspecific^36^. However, several important distinctions exist between these systems that constrain the analogy: bats move much faster in space than fish or frogs and their emission rates exhibit a broader dynamic range, making a neighboring bat’s next call emission far less predictable than is the case for a chorusing frog or electric fish. Also, the fish and frogs’ strategies are adequate only for pairs or trios, but generally don’t extend to the larger, denser social conditions that bats commonly face.

Notably, Brush and Narins^26^ recognized a similarity between the timing algorithms used by chorusing frogs and those emerging with the development of artificial communication systems. When multiple users randomly access a shared communication channel, the rate of mutual interference (I) increased following a power function (I = rt^n^) defined by the number of users (n), signal durations (t) and average transmission rates (r)^37^. The interference problem is compounded if users immediately resend signals lost due to mutual interference. Consequently, Abramson^37^ showed that as the numbers of users sharing the communication channel increases, the exponential rise in interference can only be mitigated if all users adopt a probabilistic delay algorithm for resending packets lost to interference. These so-called “back-off” algorithms effectively slow transmission rates proportional to the number of users, despite imposing constraints on individual transmission rates, and ultimately optimize information flow for all users relative to the random-access condition. Chorusing frogs weren’t faced with the same degree of challenges confounding new technologies in wireless communications and Internet trafficking, but the algorithms developed to ease congestion in artificial communication networks were nevertheless applicable to chorusing frogs^26^. Building upon this idea, we investigated whether echolocating bats might display vocal behaviors resembling the probabilistic transmission-delay algorithms commonly used in artificial communications networks to optimize their sonar performance in crowded social contexts^23^,^37^.

Working with crawling bats, we found evidence that the bats appeared to be following a back-off algorithm resembling those used in artificial communication systems^23^,^37^, but we fell short of demonstrating whether or not flying bats used this strategy while performing challenging navigational tasks, precisely when the behavior should matter the most. To test this, we flew bats through obstacles alone and while listening to artificial playback (PB) of other bats flying though the obstacles. We hypothesized that bats flying in social settings optimize sonar performance by decreasing their emission rates, counterintuitively reaping a net increase in information flow by emitting fewer pulses. This led to several predictions: (1) flying bats produce fewer pulses per second when flying in pairs than when alone, (2) interference rates affect sonar-based navigational performance, and (3) echolocation of other bats causes the reduction in emission rate, not their physical presence.

## Results

### Emission Rates Decreased in the Presence of Playback of a Simulated Bat

**Figure 1:**
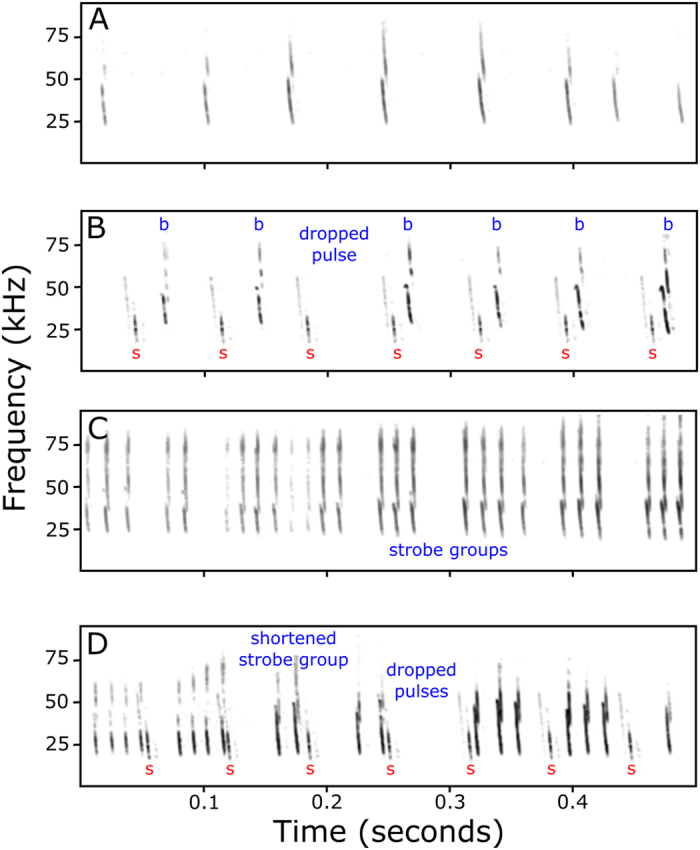
Representative spectrograms of echolocation sequences of a bat, *Tadarida brasiliensis*. (**A,B**) Pulse sequences through an open flight room (**A**) in silence and (**B**) while hearing playback (PB) of an artificial stimulus mimicking the echolocation of another bat. (**C,D**) Pulse sequences through a maze of thin ropes (**C**) in silence and (**D**) with PB. The playback stimulus is demarcated with a red “s” and the echolocation pulses of the bat with a blue “b”.

**Figure 2:**
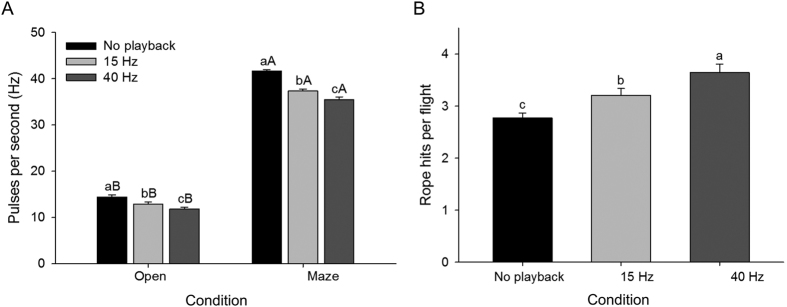
Playback of an artificial stimulus at different rates reduced echolocation rates and navigational performance of bats, *Tadarida brasiliensis.* (**A**) Echolocation emission rate (mean + 1 SEM, n = 10 bats) decreased in the presence of playback in an open room and while flying through a 6 row × 5 rope maze. (**B**) Navigational performance (n = 10, +1 SEM), measured as number of hits made with ropes in the maze, declines in the presence of playback. Different lowercase letters among playback conditions and different uppercase letters between room conditions (open vs. maze) were significantly different according to Tukey post hoc test.

To test if flying free-tailed bats optimize their echolocation performance by decreasing their emission rates, we recorded ten bats under three conditions: (1) one bat flying alone within the open flight room, (2) a simulated group with one bat flying alone with playback of artificial echolocation calls (PB) at a rate of 15 pulses per second (Hz) simulating other bats in the room, and (3) a simulated group with PB at 40 Hz. Solitary bats flying unobstructed in the open condition emitted evenly timed pulses (Fig. 1A, Supplementary Fig. S2B). Emission rates were significantly lower when the same bats flew in the presence of the PB, simulating the presence of another bat (Fig. 2A; *F*_2,29_ = 10.00, *p* < 0.001). There was a 15% reduction in emission rate with PB compared to a bat flying alone (Fig. 2A).

### Interference Degraded Navigational Performance

Bats were challenged to fly through thin nylon rope obstacles (6 rows × 5 rope maze) in the flight room, which provided a means for ensuring that biosonar behavior was measured while performing a navigational task and also provided a mechanism for assessing sonar-guided navigation performance by counting the number of contacts with ropes (hits) per flight. There was a significant increase in their emission rates through the maze compared to the open flight room (Fig. 2A; room *F*_1,58_ = 4040.722, *p* < 0.001; PB *F*_1,58_ = 74.229, *p* < 0.001; room*PB *F*_1,58_ = 12.337, *p* < 0.001). The increase in emissions when flying in the maze is achieved by emitting “strobe groups”^2^, pulses in bursts of 2–4 evenly timed pulses per grouping (Fig. 1C, Supplementary Fig. S2B). The average number of pulses per strobe group increased with rope density to plateau at four pulses per strobe group, giving rise to a maximum sustained pulse rate of 41.63 ± 0.34 Hz (Supplementary Fig. S2A).

Emission rates were significantly decreased for bats flying through the maze when in simulated pairs with PB at 15 or 40 Hz compared to when flying alone in the maze (Fig. 2B; *F*_2,27_ = 48.01, *p* < 0.001). There was also a significant reduction in navigational performance in pairs (*F*_2,27_ = 11.08, *p* < 0.001), with bats hitting more ropes in the maze with an increase in PB rate (Fig. 2B).

### Bats View Other Bats as Obstacles, but Decrease Emission Rates in Response to Echolocation

**Figure 3:**
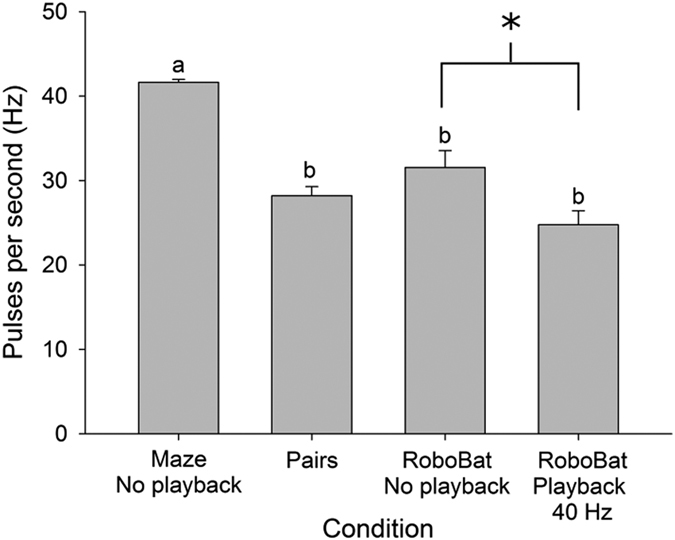
Comparison of echolocation emission rates. Bats (n = 10, +1 SEM), *Tadarida brasiliensis*, flying in four conditions: a maze of thin ropes with no playback, pairs of bats flying in open space towards each other, a single bat flying with RoboBat in silence and with playback at 40 Hz. RoboBat was a mechanical bat with flapping wings that had the same aspect ratio as *T. brasiliensis*, but did not echolocate. Conditions with different letters were significantly different according to Tamhane T2 post hoc test. *Indicates significant differences according to repeated measures ANOVA.

To test if calls of other bats caused the reduction in emission rates independent of their physical presence we compared bats’ sonar performance among three conditions: (1) two individuals flying towards each other in an open room; (2) flying alone in a room paired with tethered RoboBat, a non-echolocating robotic bat flapping its wings (Supplementary Fig. S4), without PB; and (3) flying around RoboBat while hearing 40 Hz PB. When bats flew in pairs and with RoboBat, they emitted pulses at a rate approaching, but below their maximum potential (Supplementary Figs S2A and S3), ~42 pulses per second when in the maze alone (*F*_3,36_ = 27.01, *p* < 0.001; Fig. 3). All individuals flying with RoboBat in the presence of PB emitted significantly fewer pulses than when flying around RoboBat with no PB (Wilks’ *λ*_1,9_ = 34.64, *p* < 0.001; Fig. 3 and Supplementary Fig. S3).

### Flight Paths Unaffected by Playback

**Figure 4:**
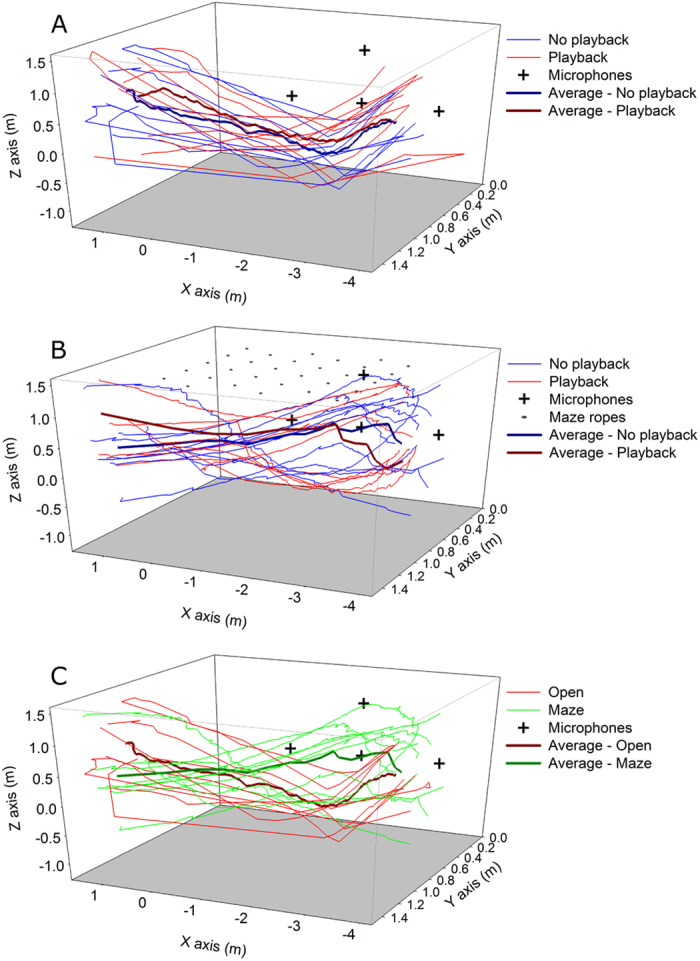
Flight paths of an individual bat, *Tadarida brasiliensis*, through the flight room in different conditions. (**A,B**) Comparison of flight paths when presented with playback of an artificial playback stimulus (n = 10) versus no playback (n = 10) in (**A**) an open room and (**B**) through a 6 row × 5 rope maze. (**C**) Example comparison of flight paths in open versus maze conditions without playback. In all figures, bold lines are the average flight path for each condition.

We reconstructed flight paths of bats with recordings from a four-microphone array to evaluate if bats altered their flight paths in response to PB. As expected, bats flew significantly slower through the maze (4.13 ± 0.12 m/s) than through the open flight room (4.92 ± 0.15 m/s; *F*_1,22_ = 16.95, *p* < 0.001), but there was no significant difference between flight path distances (open 8.10 ± 0.22 m, maze 8.29 ± 0.15 m; *p* = 0.485; e.g., Fig. 4C). Playback caused no significant change in flight speeds (open p = 0.426, maze *p* = 0.429) or total path distance (Fig. 4A,B; open *p* = 0.441, maze *p* = 0.262). There were no significant differences in flight path position in the room, width (Y-axis) and height (Z-axis), among acoustic and room conditions (all *p* > 0.1; Supplementary Table S1; e.g., Fig. 4). Overall, there were no changes in flight trajectory associated with the observed changes in pulse emissions across conditions.

## Discussion

Our results support our hypothesis that free-tailed bats flying together decrease their emission rates to minimize the effect of mutual interference with each other. We found *T. brasiliensis* emitted fewer pulses when flying in pairs than when alone in both open and cluttered (maze) conditions. The behavior is mediated by an acoustic suppression of emission rates that appears to be reflexive, being present in all individuals tested (e.g., Supplementary Fig. S3) and has not appeared to diminish over time. We found no effect of PB on flight path, speed, or distance.

Acoustic interference from other nearby bats should degrade sonar performance, with decreased navigational performance because of loss of information from acoustic interference, but to our knowledge this has never actually been observed. Our results demonstrate that hearing PB mimicking the emissions of another nearby bat significantly decreased flight performance through a simple maze. Amichai *et al*.^16^ found a slight reduction in obstacle avoidance through a cluttered (two rows of ropes) environment with jamming PB, but did not evaluate this at different PB rates. Increasing PB rate from 15 to 40 Hz was expected to increase interference rates because of a greater relative proportion of emitted pulses that overlapped with PB pulses. Higher interference rates were expected to degrade sonar-guided navigation performance even further, and this was confirmed by the observation that the bats made significantly more rope collisions per trial at the higher PB rate (Fig. 2B). This illustrates that relatively modest reductions in emission rates spread across multiple bats could provide tangible benefits to neighboring conspecifics.

Bats are known to increase emission rates in cluttered environments (e.g.^3^,^38^ and Fig. 2A open vs. maze with no playback), however computational modelling suggests that bats can improve sonar efficiency (the proportion of unambiguous echoes received per pulse emitted) by decreasing their emission rates in a social setting^23^, counterintuitively gaining more information by emitting fewer pulses when in a group. Theoretically, it should be mutually advantageous for bats to reduce the probability of overlapping echoes by emitting fewer pulses, but it was not clear how bats would balance this with the need to acquire information more rapidly in a cluttered environment by emitting more pulses. Our results indicate that they do both simultaneously, increasing emission rates in cluttered settings, while also reducing emission rates when in the presence of other bats. Foraging bats have been shown to view nearby conspecifics as obstacles^21^ and the attention hypothesis predicts that bats will adjust their call parameters to evaluate the nearby conspecific^39^. *Rhinopoma microphyllum* adjusted its echolocation in response to conspecifics as if they were background objects rather than showing any spectral jamming avoidance response^21^. The physical presence of RoboBat, which we used here to represent a single, non-echolocating bat, caused an increase in emission rate, similar to what we observed when bats flew in pairs. Once PB was added to mimic the echolocation of RoboBat, emission rates were reduced, indicating that the two different responses are being summed in the bat’s neurocircuitry controlling pulse emissions: obstacle avoidance triggers an increase in emission rate while hearing the emissions of other bats suppresses emission rates, with the two factors integrating in the bat’s brain to produce an intermediate behavioral output.

Obrist^9^ observed that bats in the field sometimes increased the time intervals between succeeding pulses when other bats were in the vicinity, and similarly, Chiu *et al*.^20^ observed *Eptesicus fuscus* omitted or postponed calls when flying close together in a lab setting. In the field it wasn’t possible for Obrist to discern whether or not observed changes in emission rates were indirectly related to changing flight path or speed, but by using a microphone array in a laboratory setting Chiu *et al*. provided reliable evidence that the changes in pulse timing were attributable to social context. Similarly, Jarvis *et al*.^36^ discovered that *T. brasiliensis* were less likely to emit pulses immediately following stimulus presentation, postponing subsequent emissions for at least 60 ms.

There is a tight mechanical pairing between wing beats and the respiratory cycle, with pulses only being emitted during the expiratory phase of the wing beat cycle^40^. Bats flying in the open flight room when in the presence of PB decreased their mean emission rates not by increasing the mean inter-pulse interval, but rather by sporadically omitting single pulses that led to an irregular overall emission pattern (Fig. 1B). Bats flying through the maze irregularly omitted entire strobe groups and/or reduced the number of pulses per strobe group (Fig. 1D). Our findings differed from those of Amichai *et al*.^16^, as they found *Pipistrellus kuhlii* decreased search phase inter-pulse intervals in the presence of jamming PB and increased emissions from one to two pulse strobe groups per wingbeat^16^. This distinction could be accounted for by the different test conditions and experimental design, or may be further evidence of species-specific differences in how bats respond to acoustic interference. There is evidence that some bats may cease calling and eavesdrop to exploit signals of their conspecifics^20^,^24^. We did not test for eavesdropping explicitly; we flew pairs of bats towards each other, in converging flight, which is an orientation that Chiu *et al*.^20^ did not find silent periods of eavesdropping, but we still found reductions in emission rates with this configuration. By dropping pulses the bats may have been able to reduce overall interference rates in an ongoing, probabilistic fashion.

Both Barber *et al*.^41^ and Chiu *et al*.^20^ show evidence of increased inter-pulse intervals in the presence of external auditory stimuli and they predicted that this was to reduce interference while processing both passive and active acoustic cues. However, we and Jarvis *et al*.^23^ found lower emission rates resulted from dropped pulses or strobe groups rather than increased inter-pulse intervals and this behavior was demonstrated by both bats when flying in pairs. This implies a mechanism for reducing interference rather than shifting focus to the other acoustic cues.

It appears that this suppression is a cooperative behavior, as each individual incurs the cost of reducing their emission rates which confers a benefit to the group. However, we argue that this mechanism supports selection at the individual level because the individuals reap reduced interference, which in turn leads to improved navigational and foraging performance while flying in denser social groups (Fig. 2B). The ability to roost and fly in dense groups of conspecifics may offer several benefits, including reduced predation risk and increased access to limited resources (caves and day roosts), resulting in a by-product mutualism among individuals^42^. Previous studies observed that every bat tested performed this behavior reflexively^23^,^36^, suggesting that cheating might be constrained by some form of neural hard-wiring. Not all species of bats are equally social, and few are as highly gregarious as *T. brasiliensis,* nor so dependent on a surprisingly limited number of caves possessing the ideal combination of temperature, humidity, and size requirements^43^. We predict that this behavior is an adaptation for high-density social groups and may be more prevalent in bat species forming large colonies. The mechanism revealed here of reducing emission rate may be better suited to low-duty cycle bats rather than species with high-duty cycle emissions. *Tadarida brasiliensis* is a low-duty cycle species that emits short, broadband FM sweeps, typical of many aerial hawking species. High-duty cycle species with constant-frequency pulses are thought to be less sensitive to overlapping temporal interferences^44^ and may not gain the same advantage from decreasing emission rates.

In conclusion, we found that flying bats display evidence of mutual suppression of echolocation and we hypothesize that this temporal modification improves their sonar efficiency, leading to improved navigational performance in social settings.

## Methods

### Animals and facilities

We used 30 wild-caught male and female free-tailed bats, *T. brasiliensis*. Animals were group-housed in an artificial habitat at Texas A&M University (TAMU) with a reversed light cycle. All experiments were carried out according to the National Institutes of Health guidelines^45^ and were approved by the TAMU Institutional Animal Care and Use Committee (AUP# 2014–0090). Bats were trained daily for at least a month to reliably fly in an open room between two platforms at opposite ends of the flight room when released by their handler. During all experiments, the bats began the trial perched on a handler’s hand at one end of the flight room and would fly to the platform at the opposite end of the flight room when cued with the playback of a social call, whereupon they received a food reward (mealworm) from a second handler. The experimental flight room was L 6 m × W 1.5 m × H 3 m, completely covered in sound-absorbing 4-inch acoustic foam. The flight room was dark for all experiments, except for infrared (IR) lights and handlers used red headlamps when handling the bats.

### Experimental design

To test if flying bats decreased their emissions in the presence of other echolocating bats, we measured their biosonar emission rates and patterns under four experimental conditions; (1) while flying across an open space with and without PB, (2) flying through a rope maze with and without PB, (3) flying across the open space at the same time as another bat, and (4) flying across an open space while maneuvering around an artificial bat with and without PB (Supplementary Table S1). Trial order was randomized with regard to PB stimulus conditions.

### Acoustic recordings and playback

The flight room was equipped with five microphones and four speakers. The bat’s calls were recorded with a condenser microphone (CM16, Avisoft Bioacoustics, Berlin, Germany) and digitized with a multifunction analog-to-digital converter (X Series, National Instruments, Austin, TX) with recording parameters set by the multichannel recording software Avisoft-RECORDER to 192 kHz sampling rate, 16 bit resolution. This microphone was positioned in the center of the room, 0.6 m above the floor on a tripod, pointing upwards. Pulses were also recorded using a custom-built microphone array composed of four omnidirectional electret microphones (FG-23329, Knowles Electronics, Itasca, IL) along the lateral wall, with three microphones positioned linearly 1.3 m above ground and 1.6 m apart and the fourth was 1 m above the middle microphone (Fig. 4, Supplementary Fig. S1).

The PB stimulus was a downward-sweeping frequency-modulated 5 ms pulse with 40 kHz bandwidth, ranging from 18 to 58 kHz (e.g., Fig. 1B,D), presented at a rate of either 15 or 40 Hz (typical emission rates for bats flying through either an open space or maze from preliminary data). Stimuli were digitally constructed with Real-time Processing Visual Design Studio software (RPvdsEx v. 70, Tucker-Davis Technologies (TDT), Alachua, FL). Previous experiments showed that the bat’s response to this stimulus mimicked their responses to naturalistic sonar. The analog signal was generated by a TDT System III RX6 real-time processor piped into a commercial amplifier (STR-DE598, Sony, Park Ridge, NJ), which powered four 1-inch square frame tweeters (model # BC25SC55-04, Tymphany HK Ltd, Sausalito, CA). There were two tweeters in the middle of each lateral wall, each pointing in opposite directions at 45°. The primary objective for these experiments was to make sure PB was always projected towards the bats as they approached the center of the room and would be audible throughout the entire flight room, regardless of the bat’s position or orientation within the space. Sounds from each speaker would have arrived at the bat at slightly different, but overlapping time windows creating perceived stimulus durations up to 2.2 ms longer than what was projected (varying with bat position). The stimulus set would have likely been perceived as emanating from different sources, but we did not evaluate the impact of numbers of sources or relative orientation in this study. Since the speakers were attached to the side walls and directed towards the center of the room the stimuli emanated orthogonal to the bat’s own flight path and would therefore have been unlikely to generate echoes that the bats could have used to supplement their own navigation.

#### Emission rates

We recorded the call emission rate of ten bats under three conditions: (1) one bat flying within the open room and simulated groups with one bat flying in the room and PB at a rate of (2) 15 Hz or (3) 40 Hz (emitted from all four tweeters; Supplementary Table S1). Each individual had ten flights per condition; once across the room was counted as one flight. Emission rates in the test zone were measured by counting the total number of calls emitted within a 500 ms time frame focused around the time when the bat passed the center of the room. This time window was selected because this was when the bats were flying at a consistent speed and trajectory, and avoided the more variable pulse rates associated with takeoff and landing. Throughout, we tested if data met all assumptions of parametric statistics using Levene’s test for homogeneity of variances and Shapiro-Wilk test for normality. We then used analysis of variance (ANOVA) to test for differences in emission rate among the three conditions and Tukey post hoc test. All statistical analyses were performed in SPSS Statistics (v.22, IBM Corp., Armonk, NY).

#### Navigational performance

To test whether hearing the pulses of another bat impacted sonar performance, such that sonar-based navigational performance could be improved by reducing interference, we flew ten bats through a rope maze (20 flights per individual) in three different acoustic conditions, (1) in silence (no PB), (2) in the presence of PB at a presentation rate of 15 Hz and (3) in the presence of PB at 40 Hz (Supplementary Table S1). The maze was constructed from 1/8-inch nylon ropes suspended from a cantilever-type piezofilm vibration sensor (Minisense 100, Digi-Key Electronics, Thief River Falls, MN). The vibration sensors were connected to a DC-powered microcontroller (Arduino Uno, Rev 3, www.store-usa.arduino.cc) that was used to condition and digitize the signal. The maze consisted of six rows of five ropes each (Fig. 4B, Supplementary Fig. S1), separated by 20 cm (~5 cm larger than wingspan). The ropes were hung from the vibration sensors, and the sensor activity from each rope fed into a discreet analog input channel on the microcontroller. Digitized output from the microcontroller was then delivered to the PC via dedicated channels on the same National Instruments A/D converter being used to collect acoustic data. Since preliminary trials indicated that the bats could develop preferred strategies for navigating the maze within trials, sensor rows were mounted on tracks that could be shifted laterally, thereby allowing rearrangement of the maze between trials. We recorded the echolocation of bats flying through the maze and counted the number of calls through the test zone (described above) and counted the number of contacts with ropes (hits) per flight that were recorded by the vibration sensors. We also visually confirmed contact with ropes with IR video recordings (acA2040-90; Basler, Inc.; Exton, PA). Most individuals actively avoided the ropes, but a few individuals made no effort to avoid the ropes and were excluded from trials. We compared results among the three conditions with an ANOVA and Tukey post-hoc test. We compared emission rates between flight room conditions (open vs. maze), PB conditions (no PB, 15 Hz, 40 Hz), and room *PB interaction with a factorial ANOVA.

#### Flying bats in pairs

Pairs of trained bats (n = 5 bats, 10 pairs) were flown at the same time, released by two handlers from opposite ends of the open flight room, resulting in the two bats flying towards each other as they passed through the room. Trials were repeated until we were able to collect data from ten successful flights per pair; a paired-flight was deemed successful if both bats took flight at nearly the same time, passed each other near the center of the room (occupied the same central flight space during the same 500 ms time window) and completed their flight to the opposite landing platform. We measured mean individual emission rates per second.

#### Flying bats with RoboBat

Preliminary trials revealed two distinct sonar responses to other bats, the first being an increase in emission rates in support of obstacle avoidance and the second being a reduction in emissions triggered by hearing another bat’s sonar emissions. Using RoboBat in combination with PB afforded the opportunity to dissect the relative contributions of these two distinct behavioral reflexes on the overall biosonar performance. RoboBat had the same wing aspect ratio as *T. brasiliensis*, but with a slightly larger body size. The mechanical flapping was audible, but had no measurable effect on bat behavior and the mechanical noise did not overlap in bandwidth with the frequencies at which the bats were echolocating (Supplementary Fig. S4). RoboBat was constructed from parts of a battery-powered, animated flying bat and was tethered from the ceiling by a 1/8 inch nylon rope to hang 1.2 m below the ceiling in the center of the flight room.

We assessed the relative impacts of RoboBat’s swinging motion versus its wing flapping on real bats by having each of ten bats make ten flights past RoboBat while it was swinging with wings flapping and ten flights while RoboBat was stationary with wings flapping, both without and with PB at 40 Hz. There was no significant difference in bat call emission rates whether RoboBat was swinging or stationary (with flapping wings) so we pooled the moving and stationary data (p = 0.945). We compared emission rates of bats flying alone in the maze to bats flying in pairs and with RoboBat using an ANOVA and Tamhane’s T2 post hoc test. We also compared between RoboBat conditions, with and without PB, with a repeated measures ANOVA because we had the opportunity to use the same individuals between the two playback conditions.

### Flight paths

To test if bats altered their flight path in response to PB and experimental conditions we reconstructed flight paths of six individuals for ten flights across the following conditions: 1a) open room with no PB, 1b) open with 15 Hz PB, 2a) maze with no PB, and 2b) maze with 40 Hz PB. We reconstructed flight paths using Moonshine (custom MATLAB script, Lasse Jakobsen, University of Southern Denmark), which triangulated the time-of-arrival of each call on each of the four microphones. From these flight paths we measured the total speed and path distance (following^46^) and mean, minimum, and maximum positions in the room, width (Y-axis) and height (Z-axis) as the bats flew across the room. All bats flew the total length of the room (X-axis). We compared these measurements among conditions using multivariate ANOVA. We also compared between PB conditions using repeated measures ANOVAs.

## Additional Information

**How to cite this article**: Adams, A. M. *et al*. Suppression of emission rates improves sonar performance by flying bats. *Sci. Rep.* **7**, 41641; doi: 10.1038/srep41641 (2017).

**Publisher's note:** Springer Nature remains neutral with regard to jurisdictional claims in published maps and institutional affiliations.

## Supplementary Material

Supplementary Material

## References

[b1] Griffin D. R. Listening in the dark: the acoustic orientation of bats and men (Yale University Press, 1958).

[b2] Moss C. F., Bohn K., Gilkenson H. & Surlykke A. Active listening for spatial orientation in a complex auditory scene. PLoS Biol. 4, e79 (2006). 16509770 10.1371/journal.pbio.0040079 PMC1393756

[b3] Petrites A. E., Eng O. S., Mowlds D. S., Simmons J. A. & DeLong C. M. Interpulse interval modulation by echolocating big brown bats (*Eptesicus fuscus*) in different densities of obstacle clutter. J. Comp. Physiol. A 195, 603–617 (2009). 10.1007/s00359-009-0435-6 19322570

[b4] Schnitzler H.-U. & Kalko E. K. V. Echolocation by insect-eating bats. BioSci 51, 557–569 (2001).

[b5] Dechmann D. K. . Experimental evidence for group hunting via eavesdropping in echolocating bats. Proc Biol Sci 276, 2721–2728 (2009). 19419986 10.1098/rspb.2009.0473 PMC2839959

[b6] Dechmann D. K., Kranstauber B., Gibbs D. & Wikelski M. Group hunting-a reason for sociality in molossid bats? PLoS One 5, e9012 (2010). 20140247 10.1371/journal.pone.0009012 PMC2815777

[b7] Kerth G. Causes and consequences of sociality in bats. BioSci 58, 737–746 (2008).

[b8] Gillam E. H., Hristov N. I., Kunz T. H. & McCracken G. F. Echolocation behavior of Brazilian free-tailed bats during dense emergence flights. J. Mammal. 91, 967–975 (2010).

[b9] Obrist M. K. Flexible bat echolocation: the influence of individual, habitat and conspecifics on sonar signal design. Behav. Ecol. Sociobiol. 36, 207–219 (1995).

[b10] Bates M. E., Stamper S. A. & Simmons J. A. Jamming avoidance response of big brown bats in target detection. J. Exp. Biol. 211, 106–113 (2008). 18083738 10.1242/jeb.009688

[b11] Gillam E. H., Ulanovsky N. & McCracken G. F. Rapid jamming avoidance in biosonar. Proc Biol Sci 274, 651–660 (2007). 17254989 10.1098/rspb.2006.0047 PMC2197216

[b12] Tressler J. & Smotherman M. Context-dependent effects of noise on echolocation pulse characteristics in free-tailed bats. J. Comp. Physiol., A 195, 923–934 (2009). 10.1007/s00359-009-0468-x PMC2825556 19672604

[b13] Hage S. R., Jiang T., Berquist S. W., Feng J. & Metzner W. Ambient noise induces independent shifts in call frequency and amplitude within the Lombard effect in echolocating bats. Proc Natl Acad Sci USA 110, 4063–4068 (2013). 23431172 10.1073/pnas.1211533110 PMC3593919

[b14] Takahashi E. . Adaptive changes in echolocation sounds by *Pipistrellus abramus* in response to artificial jamming sounds. J. Exp. Biol. 217, 2885–2891 (2014). 25122918 10.1242/jeb.101139

[b15] Chiu C., Xian W. & Moss C. F. Adaptive echolocation behavior in bats for the analysis of auditory scenes. J. Exp. Biol. 212, 1392–1404 (2009). 19376960 10.1242/jeb.027045 PMC2726850

[b16] Amichai E., Blumrosen G. & Yovel Y. Calling louder and longer: how bats use biosonar under severe acoustic interference from other bats. Proc Biol Sci 282, 20152064 (2015). 10.1098/rspb.2015.2064 PMC4707756 26702045

[b17] Warnecke M., Chiu C., Engelberg J. & Moss C. F. Active listening in a bat cocktail party: adaptive echolocation and flight behaviors of big brown bats, *Eptesicus fuscus*, foraging in a cluttered acoustic environment. Brain Behav. Evol. 86, 6–16 (2015). 26398707 10.1159/000437346

[b18] Simmons J. A. . Echolocation by free-tailed bats (*Tadarida*). J. Comp. Physiol. 125, 291–299 (1978).

[b19] Ulanovsky N., Fenton M. B., Tsoar A. & Korine C. Dynamics of jamming avoidance in echolocating bats. Proc Biol Sci 271, 1467–1475 (2004). 15306318 10.1098/rspb.2004.2750 PMC1691745

[b20] Chiu C., Xian W. & Moss C. F. Flying in silence: echolocating bats cease vocalizing to avoid sonar jamming. Proc. Natl. Acad. Sci. USA 105, 13115–13120 (2008). 10.1073/pnas.0804408105 PMC2529029 18725624

[b21] Cvikel N. . On-board recordings reveal no jamming avoidance in wild bats. Proc Biol Sci 282, 20142274 (2015). 25429017 10.1098/rspb.2014.2274 PMC4262180

[b22] Cvikel N. . Bats aggregate to improve prey search but might be impaired when their density becomes too high. Curr. Biol. 25, 206–211 (2015). 25578909 10.1016/j.cub.2014.11.010

[b23] Jarvis J., Jackson W. & Smotherman M. Groups of bats improve sonar efficiency through mutual suppression of pulse emissions. Front. Physiol. 4, 1–9 (2013). 23781208 10.3389/fphys.2013.00140 PMC3680708

[b24] Lin Y. & Abaid N. Modeling perspectives on echolocation strategies inspired by bats flying in groups. J. Theor. Biol. 387, 46–53 (2015). 26386143 10.1016/j.jtbi.2015.09.007

[b25] Brumm H. Signalling through acoustic windows: nightingales avoid interspecific competition by short-term adjustment of song timing. J. Comp. Physiol., A 192, 1279–1285 (2006). 10.1007/s00359-006-0158-x 16924503

[b26] Brush J. S. & Narins P. M. Chorus dynamics of a neotropical amphibian assemblage: comparison of computer simulation and natural behavior. Anim. Behav. 37, 33–44 (1989).

[b27] Egnor S. E., Wickelgren J. G. & Hauser M. D. Tracking silence: adjusting vocal production to avoid acoustic interference. J. Comp. Physiol., A 193, 477–483 (2007). 10.1007/s00359-006-0205-7 17242881

[b28] Ficken R. W., Ficken M. S. & Hailman J. P. Temporal pattern shifts to avoid acoustic interference in singing birds. Science 183, 762–763 (1974). 17790627 10.1126/science.183.4126.762

[b29] Knapton R. W. Intraspecific avoidance and interspecific overlap of song series in the eastern meadowlark. The Auk 104, 775–779 (1987).

[b30] Loftus-Hills J. J. Analysis of an acoustic pacemaker in Strecker’s chorus frog, *Pseudacris streckeri* (Anura: Hylidae). J. Comp. Physiol. 90, 75–87 (1974).

[b31] Moore S. W., Lewis E. R., Narins P. M. & Lopez P. T. The call-timing algorithm of the white-lipped frog, Leptodactylus albilabris. J. Comp. Physiol., A 164, 309–319 (1989).

[b32] Nelson M. E. & MacIver M. A. Sensory acquisition in active sensing systems. J. Comp. Physiol., A 192, 573–586 (2006). 10.1007/s00359-006-0099-4 16645885

[b33] Planqué R. & Slabbekoorn H. Spectral overlap in songs and temporal avoidance in a Peruvian bird assemblage. Ethology 114, 262–271 (2008).

[b34] Zelick R. & Narins P. M. Characterization of the advertisement call oscilator in the frog *Eleutherodactylus coqui*. J. Comp. Physiol., A 156, 223–229 (1985).

[b35] Heiligenberg W., Baker C. & Bastian J. J. The jamming avoidance response in gymnotiod pulse-species: a mechanism to minimize the probability of pulse-train coincidences. J. Comp. Physiol. 124, 211–224 (1978).

[b36] Jarvis J., Bohn K. M., Tressler J. & Smotherman M. A mechanism for antiphonal echolocation by free-tailed bats. Anim. Behav. 79, 787–796 (2010). 20419063 10.1016/j.anbehav.2010.01.004 PMC2858338

[b37] Abramson N. in Proceedings of the November 17–19, 1970 Fall Joint Computer Conference. 281–285 (AFIPS Press).

[b38] Falk B., Jakobsen L., Surlykke A. & Moss C. F. Bats coordinate sonar and flight behavior as they forage in open and cluttered environments. J. Exp. Biol. 217, 4356–4364 (2014). 25394632 10.1242/jeb.114132 PMC4375838

[b39] Götze S., Koblitz J. C., Denzinger A. & Schnitzler H. U. No evidence for spectral jamming avoidance in echolocation behavior of foraging pipistrelle bats. Sci Rep 6, 30978 (2016). 27502900 10.1038/srep30978 PMC4977515

[b40] Speakman J. R. & Racey P. A. No cost of echolocation for bats in flight. Nature 350, 421–423 (1991). 2011191 10.1038/350421a0

[b41] Barber J. R., Razak K. A. & Fuzessery Z. M. Can two streams of auditory information be processed simultaneously? Evidence from the gleaning bat *Antrozous pallidus*. J Comp Physiol A Neuroethol Sens Neural Behav Physiol 189, 843–855 (2003). 14564468 10.1007/s00359-003-0463-6

[b42] Connor R. C. The benefits of mutualism: a conceptual framework. Biological Reviews 70, 427–457 (1995).

[b43] Davis R. B., Herreid C. F. & Short H. L. Mexican free-tailed bats in Texas. Ecol. Monogr. 32, 311–346 (1962).

[b44] Tian B. & Schnitzler H.-U. Echolocation signals of the greater horseshoe bat (*Rhinolophus ferrumequinum*) in transfer flight and during landing. J. Acoust. Soc. Am. 101, 2347–2364 (1997). 9104033 10.1121/1.418272

[b45] Council N. R. Guide for the care and use of laboratory animals. Eighth edn (National Academies Press (US), 2011). 21595115

[b46] Emrich M. A., Clare E. L., Symondson W. O. C., Koenig S. E. & Fenton M. B. Resource partitioning by insectivorous bats in Jamaica. Mol. Ecol. 23, 3648–3656 (2014). 25187923 10.1111/mec.12504

