# Supplementary material for: Suppression of emission rates improves sonar performance by flying bats
Source: Sci Rep. 2017 Jan 31;7:41641. doi: 10.1038/srep41641 (PMC5282581; doi:10.1038/srep41641)
Supplement: Supplementary Material [file srep41641-s1.pdf]

Suppression of emission rates improves sonar performance by flying bats

Amanda M. Adams, Kaylee Davis, Michael Smotherman

Supplementary material

Table S1. Room and playback conditions for each part of the Results section.

Results section	Room condition	Playback condition	No. of bats	No. of flights per bat
Emission rates	Open	No playback	10	10
	Open	15 Hz	10	10
	Open	40 Hz	10	10
Navigational performance	Maze	No playback	10	20
	Maze	15 Hz	10	20
	Maze	40 Hz	10	20
Pairs	Open	No playback	5 pairs (10 bats)	10
RoboBat	RoboBat	No playback	10	20
	RoboBat	40 Hz	10	20
Flight Paths	Open	No playback	6	10
	Open	15 Hz	6	10
	Maze	No playback	6	10
	Maze	40 Hz	6	10

Table S2. Statistical results from multivariate analysis of variance (MANOVA) for the mean, minimum, and maximum positions in width (Y-axis) and height (Z-axis) of the flight paths of bats, *Tadarida brasiliensis*. (**A**) Comparing flight paths in the open flight room versus rope maze. (**B**) Comparing two playback conditions, no playback versus playback of an artificial echolocation call stimulus. (**C**) Results for the interaction between room and playback conditions. There were no significant differences in flight path positions between conditions; analysis is considered significant at $\alpha < 0.05$.

**(A) Room**	**Width (Y-axis)**		**Height (Z-axis)**	
	$F_{1,20}$	p	$F_{1,20}$	p
Mean	0.926	0.347	0.094	0.762
Minimum	0.006	0.940	0.894	0.356
Maximum	0.367	0.552	0.330	0.572
**(B) Playback**				
Mean	0.040	0.844	0.100	0.755
Minimum	0.891	0.356	1.990	0.174
Maximum	1.185	0.289	0.199	0.660
**(C) Room*Playback**				
Mean	0.294	0.594	0.043	0.838
Minimum	0.155	0.698	0.040	0.844
Maximum	0.098	0.758	1.121	0.302

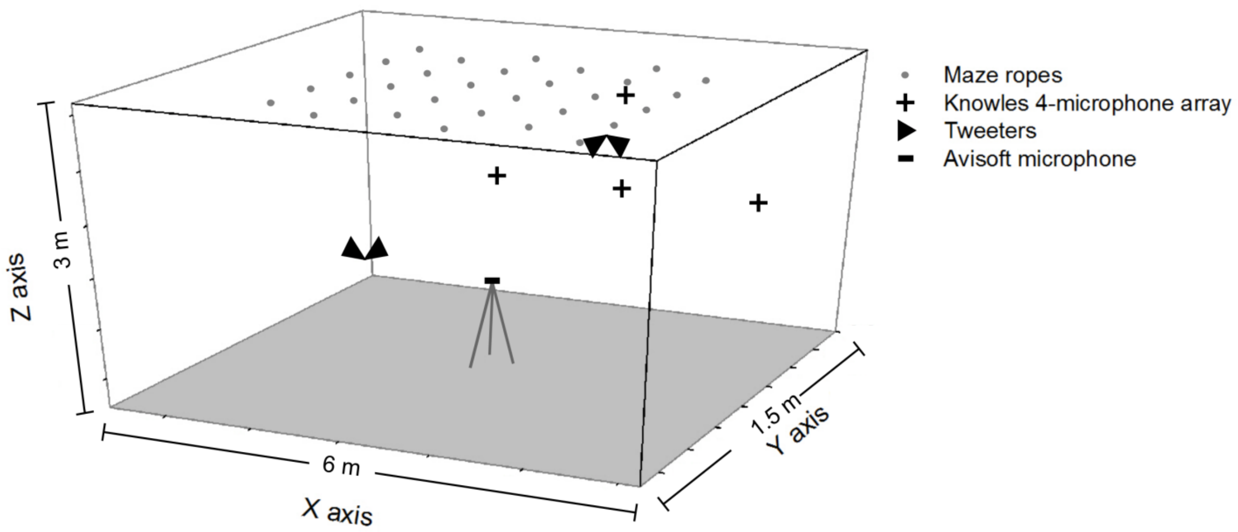

Figure S1. Flight room dimensions and equipment arrangement. An Avisoft microphone was mounted on a tripod to record bat echolocation calls for emission rates. Two tweeters were mounted on each lateral wall. A four-microphone array was used to record calls to estimate flight paths. The 6 × 5 maze had thin nylon ropes hung from ceiling to floor to evaluate navigational performance.

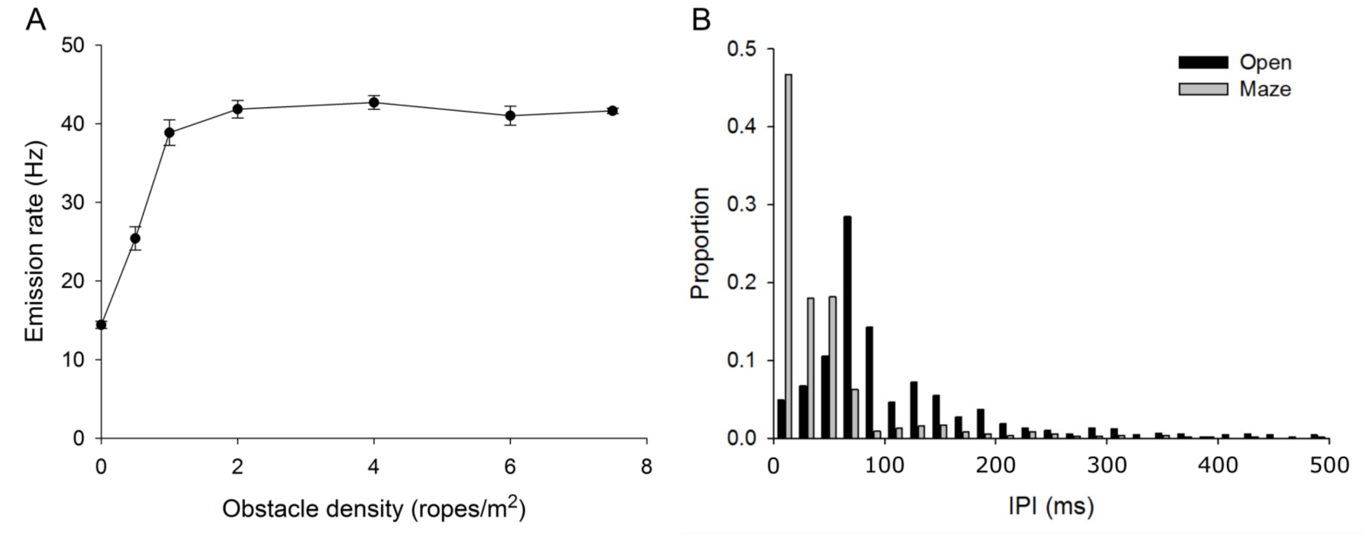

Figure S2. Pulse emission rates of bats (n = 10), *Tadarida brasiliensis*, increase when flying through an obstacle maze composed of different densities of hanging nylon ropes. (**A**) Emission rates (mean ± SEM) increase to a maximum rate with the density of ropes. Increases in pulse emissions are accomplished by adding strobe groups of 2–4 calls. (**B**) Normalized histogram of inter-pulse intervals (IPI) between calls in an open room versus the rope maze in quiet (no playback) conditions. IPIs were calculated in 20 ms bins. Strobe groups cause the shift to shorter IPIs in the maze, with the longer IPIs between the strobe groups.

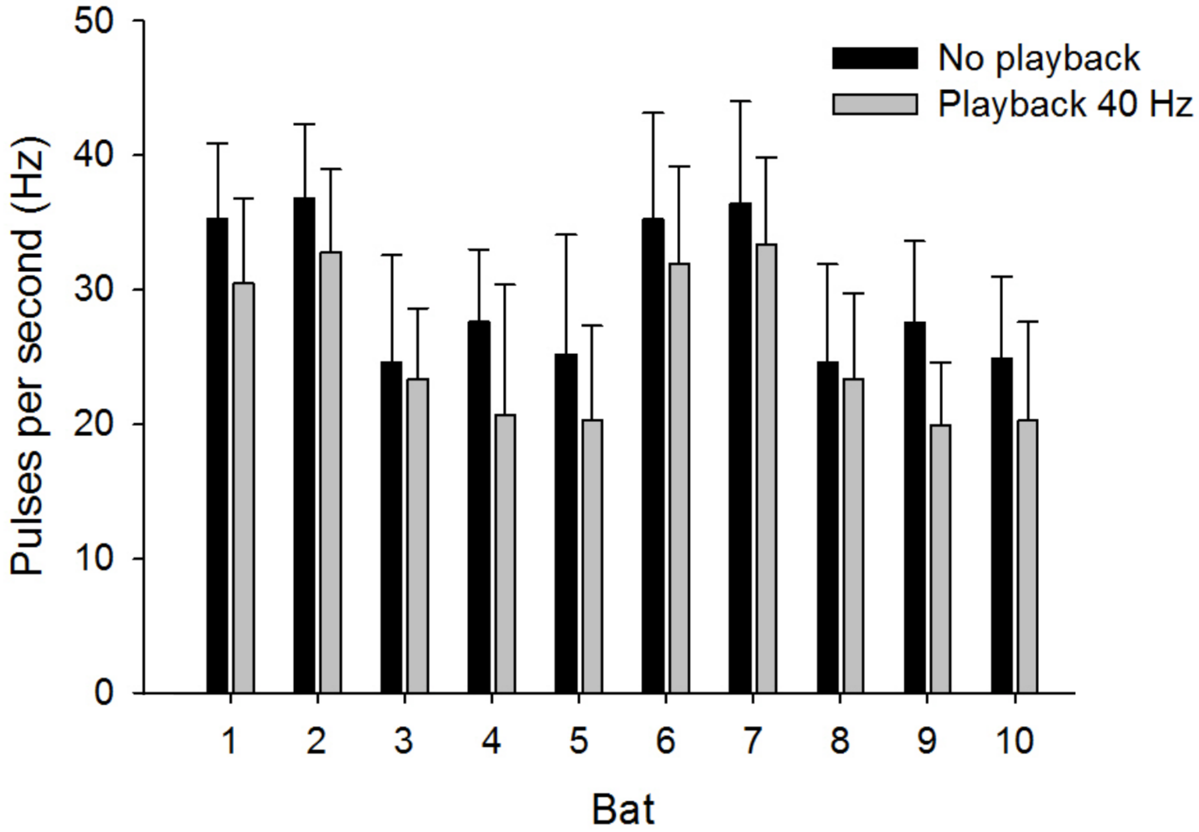

Figure S3. Individual variation of emission rates (Hz ± SD) of ten bats, *Tadarida brasiliensis*, when flying with RoboBat with and without playback. All individuals show a significant reduction of emission rate in the presence of artificial playback (repeated measures, $p < 0.001$).

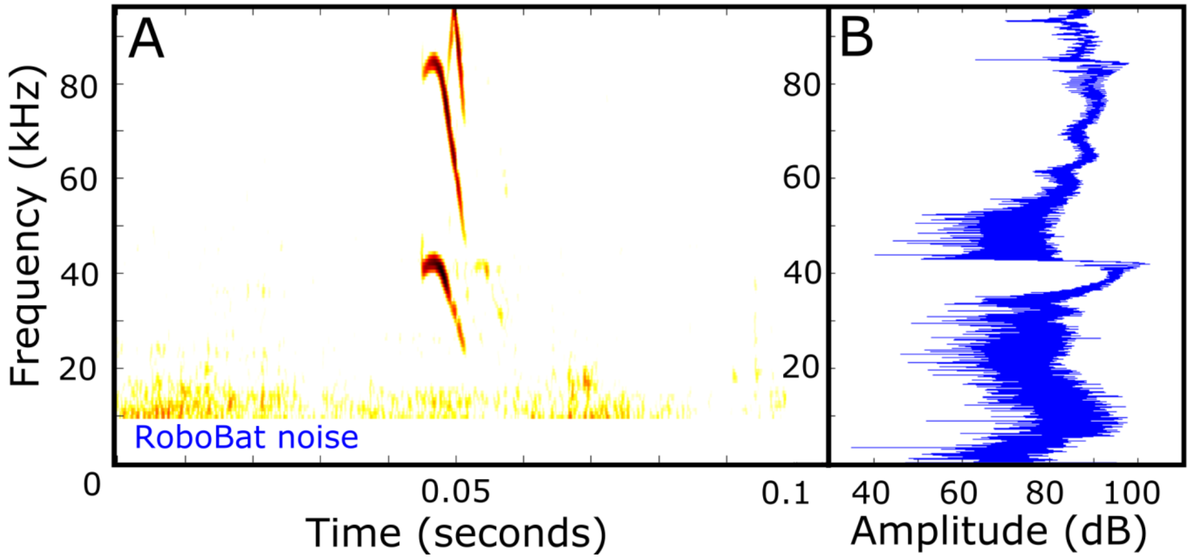

Figure S4. Mechanical noise generated by RoboBat's flapping wings (<20 kHz) is below the minimum echolocation frequency (24 kHz) of the bat, *Tadarida brasiliensis*. (**A**) Spectrogram of echolocation call and RoboBat noise. (**B**) Power spectrum showing energy distribution of sounds in (A).
